# Refolding of a Thermostable Glyceraldehyde Dehydrogenase for Application in Synthetic Cascade Biomanufacturing

**DOI:** 10.1371/journal.pone.0070592

**Published:** 2013-07-24

**Authors:** Fabian Steffler, Volker Sieber

**Affiliations:** Straubing Center of Science, Technische Universität München, Straubing, Germany; Aligarh Muslim University, India

## Abstract

The production of chemicals from renewable resources is gaining importance in the light of limited fossil resources. One promising alternative to widespread fermentation based methods used here is Synthetic Cascade Biomanufacturing, the application of minimized biocatalytic reaction cascades in cell free processes. One recent example is the development of the phosphorylation independent conversion of glucose to ethanol and isobutanol using only 6 and 8 enzymes, respectively. A key enzyme for this pathway is aldehyde dehydrogenase from *Thermoplasma acidophilum*, which catalyzes the highly substrate specific oxidation of d-glyceraldehyde to d-glycerate. In this work the enzyme was recombinantly expressed in *Escherichia coli*. Using matrix-assisted refolding of inclusion bodies the yield of enzyme production was enhanced 43-fold and thus for the first time the enzyme was provided in substantial amounts. Characterization of structural stability verified correct refolding of the protein. The stability of the enzyme was determined by guanidinium chloride as well as isobutanol induced denaturation to be ca. −8 kJ/mol both at 25°C and 40°C. The aldehyde dehydrogenase is active at high temperatures and in the presence of small amounts of organic solvents. In contrast to previous publications, the enzyme was found to accept NAD^+^ as cofactor making it suitable for application in the artificial glycolysis.

## Introduction

Chemical production is progressively turning towards more sustainable feedstocks and processes. As an alternative to petroleum, biomass can serve as a source for important chemicals such as organic solvents, e.g. n-butanol, isobutanol or ethanol. Biotechnological approaches for conversion of biomass into some of these molecules are well established by fermentation [1], [2]. However, these microbial processes show several limitations such as reduced product yield due to by-product formation, maintenance of the cells metabolism and, more importantly, low productivity and product titer due to the toxicity of the products formed [3]. One solution to these problems, which has recently found strong interest, is the elimination of the cell as production vehicle and the application of synthetic enzymatic cascades instead [4], [5], [6], [7], [8].

In Synthetic Cascade Biomanufacturing the process limits are given only by the limits of enzymes, which can be much more robust than cells. Recently we showed the prospect of this approach for the production of isobutanol and ethanol in a cell-free system [9]. This artificial, solely enzyme based cascade system shows remarkable advantages compared to fermentative processes. The enzymes used in the artificial cascade tolerate organic solvents to higher levels than cell based systems, and remain active up to 4%v/v isobutanol. At this concentration, microbial hosts are unable to survive or maintain active metabolism [1], [2], [3], [10]. In contrast to fermentative approaches, the artificial cell-free process is based on thermostable enzymes and hence is optimized to be functional at higher temperatures, leading to faster conversion and a higher product yield.

**Figure 1:**
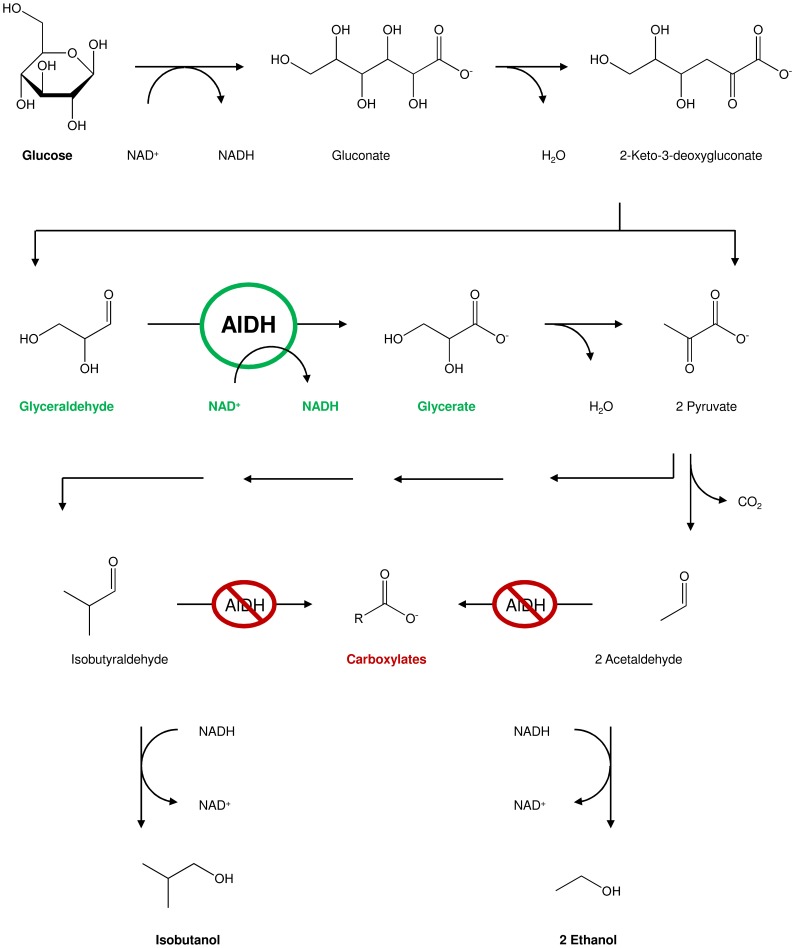
Function of glyceraldehyde dehydrogenase (AlDH) in our synthetic reaction cascade. Glucose is degraded enzymatically to pyruvate and glyceraldehyde. AlDH catalyzes the oxidation of d-glyceraldehyde to d-glycerate, which is then dehydrated to pyruvate. Ethanol and isobutanol are synthesized by further reaction steps. AlDH must not catalyze isobutyraldehyde or acetaldehyde oxidation since the irreversibly formed carboxylates are unwanted side products, thus lowering the overall yield. Important reactions are shown in detail (continuous arrows), while some reaction steps are summarized (dashed arrows).

The process is based on conversion of the substrate glucose, which represents the prevalent component of biomass. Glucose can easily be generated from different kinds of biomass. Starch is used for many biotechnological based processes by digestion of the polymer to its glucose monomers [5], [11]. Following the biorefinery concepts, glucose will be supplied by pretreatment of lignocellulosic biomass in the near future [12], [13], [14]. In the artificial biocatalytic process, glucose is enzymatically converted to the organic solvents isobutanol and ethanol (Figure 1). One key reaction in the synthetic pathway is the oxidation of d-glyceraldehyde to d-glycerate. The corresponding enzyme, glyceraldehyde dehydrogenase (AlDH), ideally should combine the following properties: high thermostability and tolerance towards solvent, high activity, acceptance of NAD^+^ as cofactor and high substrate specificity.

Screening of scientific databases revealed a number of enzymes potentially suitable for the cell-free process. Table 1 shows a selection of these AlDHs whose catalytic functions have already been shown. All enzymes have a high temperature optimum suitable for the desired temperature of the process to be in the range of 50 to 60°C [9]. The desired AlDH has to accept NAD^+^ for electron transfer since the whole enzymatic cascade is based on NAD^+^ as cofactor and accordingly all other redox enzymes are NAD^+^ dependent. NAD^+^ represents a cheaper and more stable alternative to NADP^+^ [15]. As biocatalyst for the reaction of d-glyceraldehyde to d-glycerate, the desired AlDH needs to be highly substrate specific for glyceraldehyde because other aldehydes in the substrate chain must not be targeted for oxidation (Figure 1). In a final reaction step, acetaldehyde and isobutyraldehyde are reduced to ethanol and isobutanol respectively by an alcohol dehydrogenase [16]. A competing irreversible oxidation of acetaldehyde or isobutyraldehyde by AlDH would result in Gibbs free energy release of ΔG^0^ = −50.2±5.7 kJ/mol and ΔG^0^ = −41.0±4.9 kJ/mol for oxidation to acetate and isobutyrate respectively [17]. Once formed, the carboxylates cannot reenter the pathway without activation and the production yield for organic solvents would drop dramatically.

**Table 1:** Characteristics of thermostable AlDHs for the reaction cascade shown in Figure 1.

AlDH origin	Expressionsystem	A_s_ (U/mg)	T_opt_ (°C)	Cofactor	Substrate spectrum	Reference
*Thermoplasma acidophilum*	Native	0.3	50–55	NADP^+^	Glyceraldehyde, 3-phosphoglyceraldehyde,glycolaldehyde	[36]
*Thermoplasma acidophilum*	*E. coli*	28.4	63	NADP^+^	Glyceraldehyde, 3-phosphoglyceraldehyde,glycolaldehyde	[35]
*Picrophilus torridus*	*E. coli*	10.9	63	NADP^+^	Glyceraldehyde, 3-phosphoglyceraldehyde,glycolaldehyde	[35]
*Flavobacterium frigidimaris*	Native	2.3	55–60	NADP^+^	Glyceraldehyde, various aldehydes	[52]
*Geobacillus thermoleovorans*	*E. coli*	0.1	50–55	NAD^+^	Glyceraldehyde, long chain aldehydes	[53]
*Bacillus stearothermophilus*	*E. coli*	36.3^1^	50–60	NAD^+^	Acetaldehyde, propionaldehyde,isobutyraldehyde, hexanaldehyde	[54]

A_s_: Specific enzyme activity, T_opt_: Optimum temperature of enzyme activity.

1 No activity with glyceraldehyde tested, data refers to acetaldehyde.

From the known AlDHs none appears to fulfill all requirements (Table 1). Enzymes with high substrate specificity are typically described to accept NADP^+^, whereas NAD^+^-dependent AlDHs appear to have broader substrate specificities. Since many NAD(P)^+^ dependent aldehyde dehydrogenases have a strong preference for either NAD^+^ or NADP^+^ but some are generally accepting both cofactors [18], [19], [20], we chose AlDH from *Thermoplasma acidophilum* (*Ta*AlDH) due its very high substrate specificity as most promising candidate for application in the cell-free production of ethanol and isobutanol. Here we thoroughly characterize *Ta*AlDH in light of its application and provide protocols for its production in an active state.

## Materials and Methods

### Chemicals

All chemicals were purchased in analytical grade from Sigma-Aldrich (Munich, Germany), Carl Roth (Karlsruhe, Germany), Serva and Merck (Darmstadt, Germany).

### Cloning

Codon-optimized *taaldh* gene was provided by Geneart (Regensburg, Germany); *taaldh* gene sequence was translated from protein sequence (NCBI accession number CAC11938.1). Construction of the expression vector was performed according to the protocol of Guterl *et al.* [9].

### *Ta*AlDH Production

For recombinant expression *E. coli* BL21 (DE3) (F^−^ *ompT hsdSB* (*rB* ^−^ *mB* ^−^) *gal dcm*), purchased from Novagen (Nottingham, UK), was transformed with pCBRHisC-*taaldh*, which codes for the C-terminal His-tag fusion of the protein.

Small scale protein expression was performed in shake flasks. Positive transformants of *E. coli* BL21 (DE3) were grown in auto-induction medium [21] at 37°C overnight. Cell lysis was performed with B-PER protein extraction reagent (Thermo Scientific, Ulm, Germany).

Large amounts of *Ta*AlDH -expressing cells were produced by fed-batch fermentation according to the protocol of Neubauer *et al.* [22] in a 40 L Biostat Cplus bioreactor (Sartorius, Göttingen, Germany). Defined medium was supplemented with 30 mg/L kanamycin. After inoculation, cells were grown for 24 h at 37°C and then induced with 70 mg/L IPTG. Enzyme expression was performed for 3 h, yielding 10 g/L cells (wet weight).

Cells were harvested by centrifugation at 5,000×g for 10 min at 25°C (Sorvall RC6+, Thermo Scientific), suspended in 10-fold the amount of loading buffer (200 mM NaCl, 20 mM imidazole, 2.5 mM MgCl_2_, 50 mM TRIS pH 8) containing 10 U/mL DNase I (Applichem, Darmstadt, Germany) and lysed with Basic-Z Cell Disruptor (Constant Systems, Northants, UK).

### *Ta*AlDH Purification

If not otherwise stated, purification steps were carried out at room temperature. Cell debris and protein aggregates were separated from soluble fraction by centrifugation at 30,000×g for 45 min.

After heat treatment at 50°C for 30 min, **soluble** ***Ta*** **AlDH** was further purified by nickel affinity chromatography using an ÄKTA UPC-900 FPLC-system (GE Healthcare, Freiburg, Germany). Supernatant was loaded on a HiTrap FF-column previously equilibrated with loading buffer. After a subsequent washing step, *Ta*AlDH was eluted with imidazole buffer (200 mM NaCl; 500 mM imidazole; 50 mM TRIS pH 8) at a flow rate of 5 mL/min and fractions containing *Ta*AlDH were combined. The buffer was switched to 20 mM (NH_4_)HCO_3_ with HiPrep 26/10 desalting column and *Ta*AlDH was lyophilized with an Alpha 2–4 LD Plus freeze dryer (Martin Christ, Osterode am Harz, Germany).

Prior to refolding, **insoluble** ***Ta*** **AlDH inclusion bodies** were purified to remove other insoluble materials from the pellet. First, inclusion bodies in the pellet were suspended in cleaning buffer (0.5% Triton X, 1 mM EDTA, 20 mM TRIS pH 8); the suspension was stirred for 20 min and centrifuged (30,000×g, 4°C, 45 min). After that, the inclusion bodies were washed twice with washing buffer (1 mM EDTA, 20 mM TRIS pH 8). The purified inclusion body pellets were stored at −20°C.

### Refolding *Ta*AlDH

Purified *Ta*AlDH inclusion bodies were dissolved in denaturation buffer (6 M guanidinium chloride (GdmCl), 2 mM dithiothreitol, 20 mM TRIS pH 8) at a protein concentration of 6.0 mg/mL and incubated for 1 h at 25°C.

*Ta*AlDH refolding was achieved by 30-fold dilution of the denaturation buffer and refolding yield was analyzed after dilution in different refolding buffers (20 mM sodium phosphate pH 6, 20 mM HEPES pH 7 or 20 mM TRIS pH 8) with additives (0–20% glycerol and 0–0.5 M NaCl). After incubation for 4 h at 25°C or 4°C, activities of these samples were measured under standard assay conditions (see below) and compared to the specific activity of soluble *Ta*AlDH.

Matrix-assisted refolding was performed according to the protocol of Holzinger *et al.* [23] using 0.5 M NaCl, 20% glycerol, 20 mM TRIS pH 8 as refolding buffer. 6.0 mg/mL unfolded *Ta*AlDH inclusion bodies were loaded on HiTrap FF-column and washed with denaturation buffer with a flow of 5 mL/min. *In vitro* refolding was performed by gradually increasing the refolding buffer concentration from 0–100% in 90 min. The treated enzyme was purified as described above for soluble purification.

### Gel Filtration

Soluble and refolded *Ta*AlDH were analyzed via gel filtration on a Superdex 200 column (GE Healthcare). Calibration of the column was performed according to the manufacturer’s instructions using thyroglobulin (M = 670 kDa), ferritin (M = 440 kDa), catalase (M = 232 kDa), γ-globulin (M = 158 kDa), aldolase (M = 158 kDa), ovalbumin (M = 44 kDa) and myoglobin (M = 17 kDa) as molecular mass standards; blue dextran was used as void volume marker. The elution of proteins was detected by UV absorption. The elution volume V_e_ was correlated with the molecular mass M by
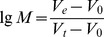



where V_t_ and V_0_ are the total volume and the void volume of the column, respectively. V_e_ of *Ta*AlDH was determined by injection of 50 mg lyophilized *Ta*AlDH, dissolved in 10 mL sample buffer (20 mM TRIS pH 8). M of *Ta*AlDH oligomers were determined from elution profile V_e_ using the calibration described above.

### Protein Determination

*Ta*AlDH solubility and inclusion body purification steps were monitored by SDS-PAGE [24]. Insoluble fractions were solubilized in 8 M urea or homogenized in cleaning or washing buffer before SDS-PAGE sample preparation. Bradford assay [25] was used for protein quantification during purification, with BSA as standard. *Ta*AlDH inclusion bodies were quantified between purification steps from homogenized samples. The molecular absorption coefficient of *Ta*AlDH at 280 nm was calculated by ProtParam tool to be ε = 82.28 mM^−1^·cm^−1^ (www.expasy.org) [26] and applied to determine the exact protein concentration in further characterization experiments [27].

### Activity Assays

Unless otherwise stated, measurements were performed at 50°C. *Ta*AlDH activity was determined spectrophotometrically by measuring the rate of NAD^+^/NADP^+^ reduction at 340 nm (ε_NADH/NADPH_ = 6.22 mM^−1^·cm^−1^) in flat bottom microtiter plates (Greiner Bio-One, Solingen, Germany) with a Fluostar Omega Photometer (BMG Labtech, Ortenberg, Germany). One unit of activity was defined as reduction of 1 µmol of cofactor per minute. Standard assay mixtures (total volume 0.2 mL) contained 1 mM d-glyceraldehyde, 2 mM NAD^+^ and appropriate amounts of enzyme in 100 mM HEPES pH 7.

The **cofactor acceptance** of *Ta*AlDH for NAD^+^ and NADP^+^ was determined in 100 mM HEPES pH 6.2. The assay contained 0.02 mg/mL refolded *Ta*AlDH, 5 mM d-glyceraldehyde and either NAD^+^ (0–75 mM) or NADP^+^ (0–0.24 mM). NAD^+^ was soluble in all concentrations in 100 mM HEPES pH 6.2 at 25°C. Apparent v_max_ and K_m_ values of the cofactors were calculated by fitting initial rate data to the Michaelis-Menten equation using Sigma Plot.

The **substrate specificity** of *Ta*AlDH for different aldehydes was determined using 4 mM NAD^+^ and 10 mM of the respective aldehyde (d-glyceraldehyde, acetaldehyde, propionaldehyde, n-butyraldehyde, pyruvaldehyde, isobutyraldehyde or d-glucose). Activity was determined at 50°C in 100 mM HEPES pH 7. The assay for d-glyceraldehyde was performed using 0.02 mg/mL refolded *Ta*AlDH and the assays with other aldehydes were performed with up to 0.45 mg/mL refolded *Ta*AlDH.

The **activity** of refolded *Ta*AlDH was tested **in presence of organic solvents**. 0.20 mg/mL *Ta*AlDH was incubated for 30 min in 100 mM HEPES pH 7 containing different organic solvents and organic solvent concentrations (0–8% v/v n-butanol, 0–9% v/v isobutanol or 0–20% v/v ethanol). After 10-fold dilution of supernatant, remaining activity was tested in respective incubation mixture.

The **solvent dependent deactivation** of *Ta*AlDH was tested at 50°C. The enzyme (0.20 mg/mL) was incubated in 100 mM HEPES pH 7 containing 0% v/v, 0.3%v/v or 3%v/v isobutanol. After different incubation times (2 min, 10 min, 30 min, 1 h, 2 h and 24 h), samples were taken and analyzed for *Ta*AlDH activity. For activity measurement, samples were diluted 10-fold to reduce the amount of enzyme in the measurement. Dilution was into identical buffer and solvent conditions (0% v/v, 0.3% v/v or 3% v/v isobutanol, respectively).

To analyze **enzyme reactivation**, *Ta*AlDH was diluted from 3% v/v isobutanol to 0.3% v/v isobutanol. For this, 0.2 mg/mL *Ta*AlDH was incubated in 100 mM HEPES pH 7 containing 3% v/v isobutanol for 30 min before 10-fold dilution with 100 mM HEPES pH 7 (giving a final concentration of 0.3% v/v isobutanol during activity measurement). To analyze the **dependence of enzyme reactivation on time of inactivation**, reactivation was also tested accordingly after different times (2 min, 10 min, 30 min, 1 h, 2 h and 24 h) of inactivation at 3% v/v isobutanol. To analyze the **dependence of enzyme reactivation on time of reactivation**, activity was also measured after different times (2 min, 10 min and 30 min) of incubation after dilution to 0.3% v/v isobutanol.

### Circular Dichroism

Circular dichroism (CD) spectra were recorded at room temperature (25±0.1°C) on a Jasco J600 spectropolarimeter (Jasco, Milan, Italy). Far-UV CD of soluble *Ta*AlDH (0.36 mg/mL) and refolded *Ta*AlDH (0.27 mg/mL) in sample buffer were measured in cuvettes with layer thickness of 0.2 cm. Near-UV CD of soluble *Ta*AlDH (0.34 mg/mL) and refolded *Ta*AlDH (0.37 mg/mL) in sample buffer and unfolded *Ta*AlDH (4.51 mg/mL) in denaturation buffer were measured in cuvettes with layer thickness of 1 cm. The observed ellipticity at wavelength λ (θ_λ_) was recorded with Jasco J600 spectropolarimeter using Spec-Man II software. The mean amino acid residue ellipticity [θ]_MRW,λ_ of *Ta*AlDH was calculated with
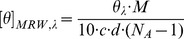
where M is the molecular mass of the protein with 56.4 kDa for glyceraldehyde dehydrogenase, c is the enzyme concentration in mg/mL, d is the path length of the cell in cm and N_A_ is the number of amino acids in the protein [28]. The secondary structure contents of the proteins were predicted from [θ]_MRW,λ_ of far-UV CD spectra by the neural-network-based algorithm K2d from Dichroweb using reference protein dataset RDB3 [29].

### Fluorescence Spectroscopy

Unfolding of *Ta*AlDH was monitored by fluorescence spectroscopy, using a Varioskan Flash Multimode Reader (Thermo Scientific), in Nunc-Immuno™ MicroWell™ 96 well polystyrene plates (Sigma-Aldrich). Protein unfolding by chemical denaturants was tested by dissolving *Ta*AlDH at a concentration of 0.04 mg/mL in sample buffer containing varying concentrations of GdmCl (0–6 M) and dithiothreitol (0–2 mM). After incubation for 1 h at room temperature (25±0.1°C), fluorescence emission was measured at 330 nm upon excitation at 280 nm at incubation temperature (25±0.1°C) and normalized (divided by the observed fluorescence emission maximum). For organic solvent induced protein unfolding, 0.04 mg/mL *Ta*AlDH was dissolved in sample buffer containing 0–9% v/v isobutanol and 0–2 mM dithiothreitol. After incubation for 30 min at 40±0.1°C, fluorescence emission was measured at 330 nm upon excitation at 280 nm at incubation temperature (40±0.1°C) and normalized (divided by the observed fluorescence emission maximum).

Gibbs free energy of denaturation in absence of denaturant and solvent ΔG^0^_D_ for *Ta*AlDH was calculated according to Santoro and Bolen [30], assuming a 2-state model and that protein unfolding is reversible. With pre- and postdenaturational baselines of respective folded and unfolded *Ta*AlDH giving a slope of zero the original equation was simplified to
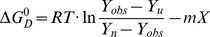
where Y_u_ and Y_n_ are the normalized fluorescence of protein in unfolded and folded state, respectively, Y_obs_ is the normalized fluorescence at denaturant concentration X, and m is related with denaturation cooperativity. Denaturation curves were fitted for parameter X to Y_obs_ by nonlinear regression using Sigma Plot.

To test the reversibility of enzyme unfolding, 1.2 mg/mL *Ta*AlDH was dissolved in denaturation buffer and diluted 30-fold in sample buffer containing varying concentrations of GdmCl (0–6 M) and dithiothreitol (0–2 mM). Moreover, 1.2 mg/mL *Ta*AlDH was dissolved in sample buffer containing 9% v/v isobutanol and diluted 30-fold in sample buffer containing 0–9% v/v isobutanol and 0–2 mM dithiothreitol. Refolding of *Ta*AlDH was monitored by fluorescence spectroscopy as described for unfolding.

### Molecular Modeling

A homology model from *Ta*AlDH amino acid sequence was created with phyre^2^ based on the crystal structure of betaine aldehyde dehydrogenase (betB) from *Staphylococcus aureus* in complex with NAD^+^ as template (RCSB PDB ID 3FG0), which had 1.85 Å resolution. The model covered 98% of *Ta*AlDH sequence and was identical to 34% [31].

## Results

### Production of Functional *Ta*AlDH

**Figure 2:**
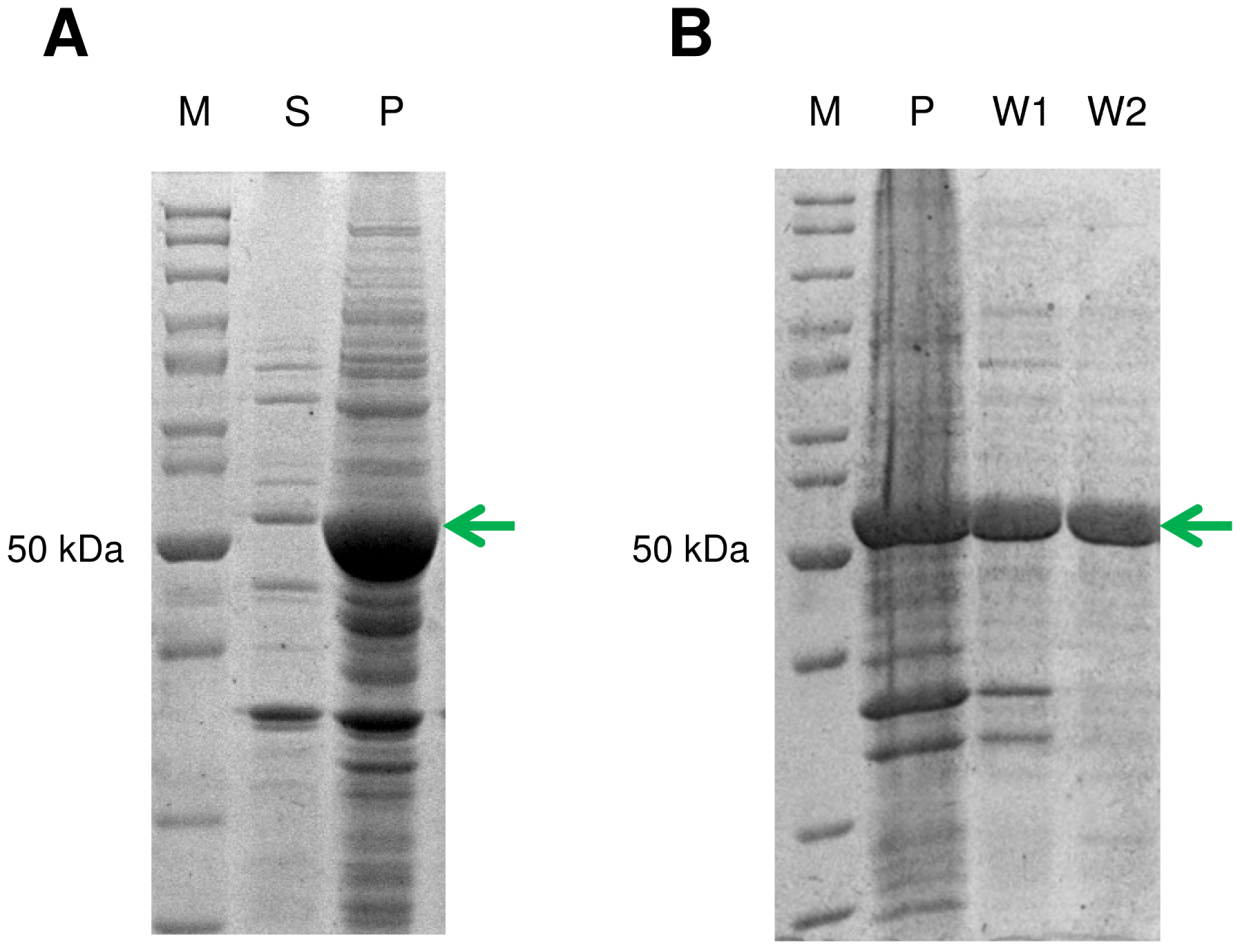
A) solubility of *Thermoplasma acidophilum* AlDH (*Ta*AlDH) and B) inclusion body purification. After recombinant expression of *Ta*AlDH in *E. coli*, soluble (S) and insoluble fraction (P) were analyzed by SDS-PAGE. *Ta*AlDH inclusion bodies from fermentation were purified with washing buffer in 2 steps (W1, W2) from insoluble fraction (P). Protein marker (M) indicates size of *Ta*AlDH (arrow).

The gene of *Ta*AlDH was synthesized and cloned into a T7 based vector system for recombinant expression in *E. coli*. This host was chosen for its advantage that target thermostable enzymes can be separated from mesophilic host proteins with a simple heat incubation step [32] and because of future protein engineering for improvement of enzyme properties [33], [34]. For its first partial characterization *Ta*AlDH had been previously recombinantly produced in *E. coli* [35]. However, no information was given on the yield of its expression. We found *E. coli* to recombinantly produce *Ta*AlDH mostly in its insoluble form. Despite efforts to improve solubility by varying *E. coli* host strains, growth medium and temperature, still over 95% of *Ta*AlDH were inactive in the form of inclusion bodies (Figure 2A). After *Ta*AlDH production via fed-batch fermentation, the soluble enzyme fraction was purified and finally lyophilized for easier storage and dosage (Table 2). Due to its low solubility, we obtained only 0.9 mg pure *Ta*AlDH from 1 L fermentation broth.

**Table 2:** Purification of soluble *Ta*AlDH from 10 g of *E. coli* cells produced in 1 L of fed-batch fermentation.

	Total volume(mL)		Protein concentration^1^(mg/mL)	Totalprotein^1^ (mg)	Volumetric activity^2^(U/mL)	Totalactivity^2^ (U)	Specific activity^1,2^ (U/mg)
**Soluble fraction**		120	2.62	314.6	n. d.	n. d.	n. d.
**Heat treatment**		120	1.22	146.7	0.002	0.23	<0.1
**Filtration**		120	1.18	141.7	0.002	0.26	<0.1
**Nickel affinity chromatography**		16	0.09	1.4	0.019	0.30	0.21
**Desalting**		20	0.07	1.3	0.015	0.30	0.22
**Lyophilization**		20	0.06/0.04	1.2/0.86	0.013	0.26	0.21/0.31

n. d.: Not determinable.

1 Protein concentration was determined with Bradford Assay/additionally after lyophilization by UV absorption spectroscopy.

2 Enzyme activity was analyzed using NAD^+^ as electron acceptor during purification.

To improve the overall yield of active protein, *Ta*AlDH inclusion bodies were used for refolding experiments. Prior to refolding, inclusion bodies were purified by two washing steps. No loss of *Ta*AlDH was observed during the washing steps and the final purity was estimated to be over 90% (Figure 2B). After dissolving purified inclusion bodies in 6 M GdmCl, different refolding conditions were tried in order to maximize the yield (Table 3).

**Table 3:** *Ta*AlDH enzyme activity after refolding purified *Ta*AlDH inclusion bodies by dilution under different renaturing conditions.

Buffer and pH	NaCl (M)	Glycerol (%)	Relative activity (%)refolded at 4°C	Relative activity (%)refolded at 25°C
Phosphate pH 6	0.5	–	<1	<1
	0.5	20	<1	<1
	–	–	<1	<1
	–	20	<1	11
HEPES pH 7	0.5	–	<1	19
	0.5	20	18	37
	–	–	<1	<1
	–	20	8	25
TRIS pH 8	0.5	–	<1	35
	0.5	20	20	51
	–	–	<1	19
	–	20	2	44

Activity after dilution in phosphate buffer pH 6 could only be detected when glycerol was supplemented and refolding took place at room temperature. Refolding of *Ta*AlDH worked best at room temperature in TRIS pH 8 with glycerol and NaCl. Here, 51% of activity could be restored compared to the specific activity of soluble *Ta*AlDH and total protein in inclusion bodies. The best refolding conditions (TRIS pH 8, 0.5 M NaCl, 20% glycerol) were applied to matrix-assisted refolding of large amounts of insoluble *Ta*AlDH (Table 4).

**Table 4:** Refolding of *Ta*AlDH from 10 g of *E. coli* cells produced in 1 L of fed-batch fermentation.

	Totalvolume (mL)	Protein concentration^1^(mg/mL)	Totalprotein^1^ (mg)	Volumetric activity^2^ (U/mL)	Totalactivity^2^ (U)	Specific activity^1,2^(U/mg)
**Insoluble fraction**	120	3.28	393.5	<0.1	<0.1	<0.1
**1^st^ inclusion body washing**	120	0.70	83.6	<0.1	<0.1	<0.1
**2^nd^ inclusion body washing**	120	0.59	70.8/49.86	<0.1	<0.1	<0.1
**Matrix-assisted refolding**	42	1.40	58.7	0.260	10.91	0.19
**Desalting**	60	0.99	59.2	0.211	12.66	0.21
**Lyophilization**	60	0.97	58.3/41.02	0.190	11.38	0.20/0.28

1 Protein concentration was determined with Bradford Assay/additionally after inclusion body washing and lyophilization by UV absorption spectroscopy.

2 Enzyme activity was analyzed using NAD^+^ as electron acceptor during purification.

After washing *Ta*AlDH inclusion bodies, they were again dissolved in 6 M GdmCl and bound via His-tag to nickel-agarose. Elution with imidazole gave a final yield of 41.0 mg from 49.9 mg protein within the inclusion bodies. Imidazole had no influence on activity. Specific activity of refolded and soluble *Ta*AlDH was comparable (0.28 U/mg and 0.31 U/mg, respectively). Total activity of lyophilized product from refolded *Ta*AlDH was 11.38 U/L, while soluble purification resulted in only 0.26 U/L. Hence, with matrix-assisted *Ta*AlDH refolding, the overall yield for total enzyme units could be increased 43-fold.

### Structural Analysis of Functional *Ta*AlDH

**Figure 3:**
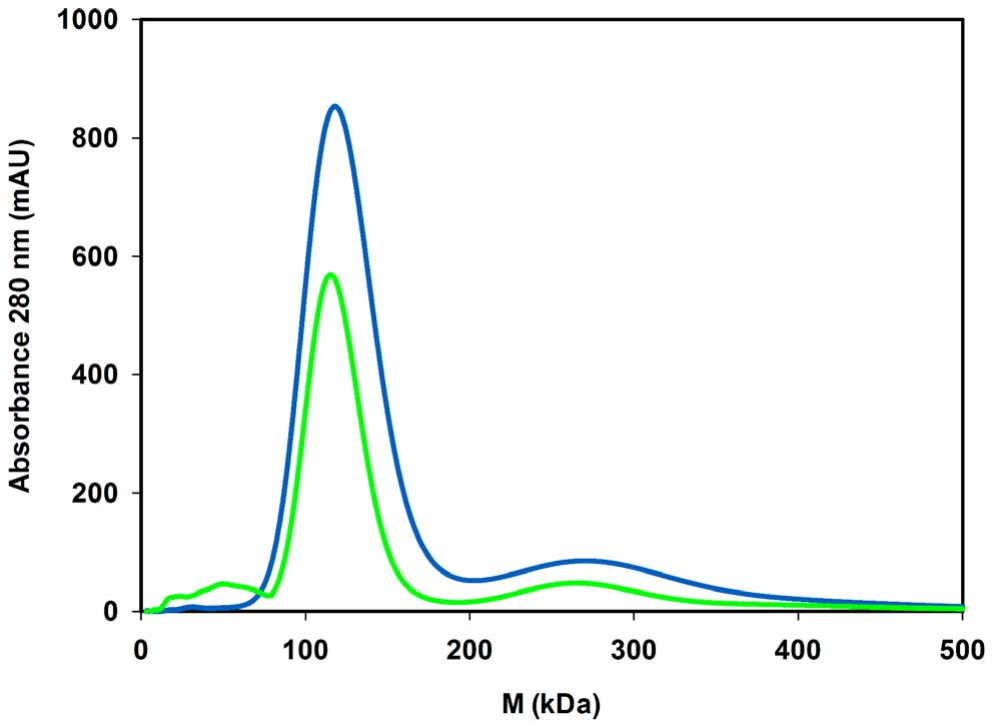
Size exclusion chromatography of *Ta*AlDH. Samples contained soluble *Ta*AlDH (blue) and refolded *Ta*AlDH (green).

Previously, native and recombinant *Ta*AlDH were reported to appear as tetramer and dimer, respectively [35], [36]. In this study, we compared oligomeric states of soluble and refolded *Ta*AlDH by size exclusion chromatography (Figure 3). Both preparations show a maximum around M = ∼120 kDa and small amounts around M = ∼260 kDa. According to the size estimation of *Ta*AlDH monomer from SDS-PAGE (Figure 2) and the molecular mass calculated from the amino acid sequence (M = 56 kDa), soluble *Ta*AlDH as well as refolded *Ta*AlDH elution contain mainly dimers with small amounts of maybe tetramers. Refolded *Ta*AlDH in addition showed the presence of very small amounts of impurities or possibly monomeric protein around M = ∼50 kDa, which after elution from the column did not show any *Ta*AlDH activity under standard assay conditions.

**Figure 4:**
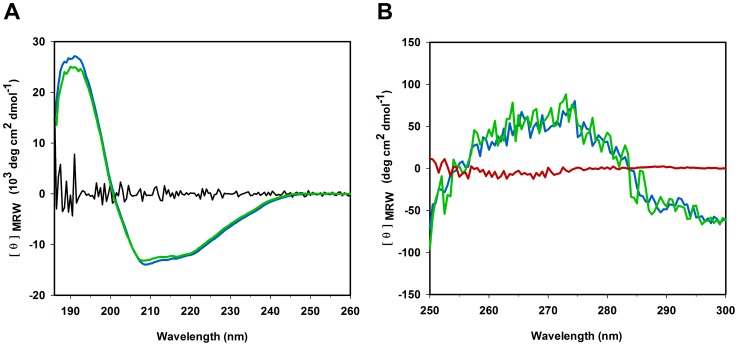
CD spectra of *Ta*AlDH. A) Far-UV CD spectrum of soluble *Ta*AlDH (blue) and refolded *Ta*AlDH (green) with standard deviation (black). B) Near-UV CD of soluble *Ta*AlDH (blue), refolded *Ta*AlDH (green) and unfolded *Ta*AlDH (red).

Refolded and soluble *Ta*AlDH were also compared by CD spectroscopy. The far-UV CD spectra of both types of *Ta*AlDH had one maximum at 192 nm and two minima at 209 nm and 220 nm. Evaluation of molar ellipticity in far-UV CD spectra indicated characteristics of ca. 37% α-helices and ca. 17% β-sheets for both soluble and refolded *Ta*AlDH. These findings are in reasonable agreement with a *Ta*AlDH homology model, which showed 40% α-helices and 20% β-sheets (Figure S1). Differences in the far-UV CD spectra between both enzymes were marginal and within standard deviation, indicating complete refolding of secondary structure with identical conformations (Figure 4A). In addition, the near-UV CD spectra of soluble protein and refolded protein were very similar (Figure 4B). All further characterization was therefore performed with refolded *Ta*AlDH.

### *Ta*AlDH Activity

**Figure 5:**
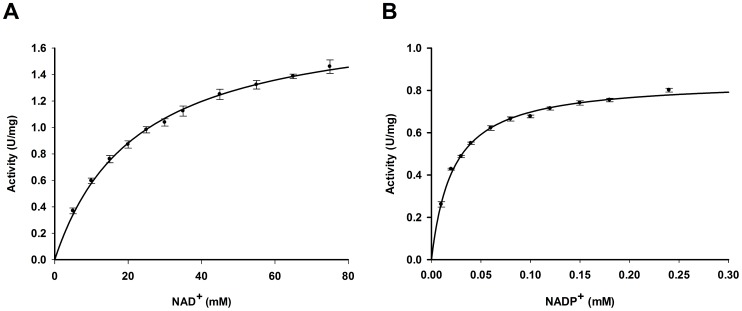
Michaelis-Menten kinetics of refolded *Ta*AlDH with cofactors A) NAD^+^ and B) NADP^+^. Reaction rates were determined in 100 mM HEPES pH 6.2 at 50°C with 5 mM d-glyceraldehyde and various concentrations of NAD^+^ or NADP^+^, respectively.

Previously, *Ta*AlDH was described as not accepting NAD^+^ as cofactor [35], [36]. Having larger amounts of protein available, we more thoroughly characterized *Ta*AlDH in the oxidation of d-glyceraldehyde to d-glycerate with both cofactors and found it to be active with NAD^+^ as well (Table 5). Rate dependencies on the cofactors followed Michaelis-Menten kinetics (Figure 5). Compared to NAD^+^, K_m_ and v_max_ values for NADP^+^ were 1000-fold lower and 2-fold lower, respectively. v_max_ determined for refolded *Ta*AlDH was lower in comparison to the earlier described recombinant enzyme, but in the range of the native enzyme [35], [36]. While still being majorly an NADP^+^ dependent AlDH, the activity was high enough at 5 mM NAD^+^, the concentration which is used in the synthetic cascade.

**Table 5:** Kinetic parameters of refolded *Ta*AlDH with different cofactors NAD^+^ and NADP^+^ at 50°C, pH 6.2.

Cofactor	K_m_ (mM)	v_max_ (U/mg)^1^	
NADP^+^	0.022±0.001	0.849±0.007	
NAD^+^	22.13±1.43	1.859±0.047	

1 0.02 mg/mL *Ta*AlDH.

Specificity of *Ta*AlDH had already been tested in previous publications using various substrates [35], [36]. However, activities with isobutyraldehyde or n-butyraldehyde, which are important regarding the solvent production cascade shown in Figure 1, have not yet been examined. Thus, these two aldehydes were tested along with other aldehydes appearing as intermediates in the cascade (Table 6). The lack of activity towards oxidation of acetaldehyde from previous publications could be confirmed. d-Glucose was tested to see whether *Ta*AlDH would accomplish the first reaction step of the synthetic cascade, but its substrate specificity was too high, resulting in activity with d-glyceraldehyde only.

**Table 6:** Substrate specificity of refolded *Ta*AlDH at 50°C, pH 7.0.

Substrate	Relative activity (%)
**d-**Glyceraldehyde	100.0
Acetaldehyde	<0.1
Propionaldehyde	<0.1
n-Butyraldehyde	<0.1
Pyruvaldehyde	<0.1
Isobutyraldehyde	<0.1
**d-**Glucose	<0.1

### The Effect of Organic Solvent on *Ta*AlDH Enzyme Activity

Within the cell-free process, participating enzymes are supposed to tolerate high product concentrations. The proper function of the enzymes must not be inhibited by the organic solvents ethanol or isobutanol. Accordingly, *Ta*AlDH activity assays were performed in presence of these molecules. Since we are expanding our tool box for cell-free production continuously, we also tested *Ta*AlDH in n-butanol.

**Figure 6:**
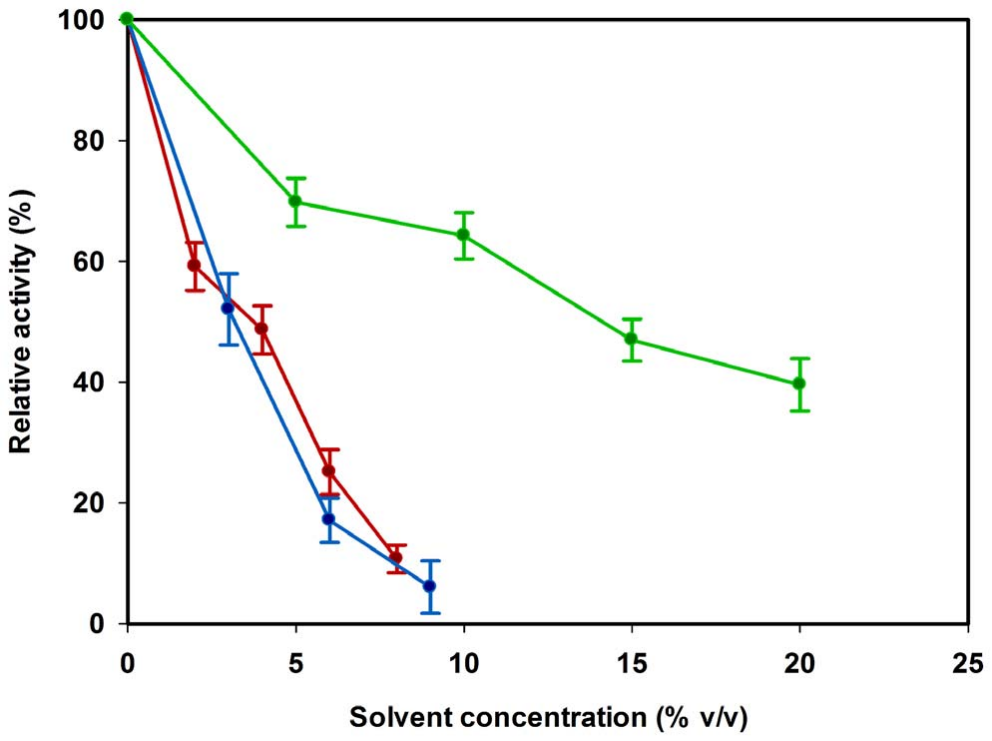
*Ta*AlDH activity in the presence of different organic solvents. Refolded *Ta*AlDH was incubated at 50°C for 30 min in 100 mM HEPES pH 7 containing various concentrations of ethanol (green), isobutanol (blue) or n-butanol (red). Remaining enzyme activity was tested at 50°C in respective incubation buffers.

The activity of *Ta*AlDH decreased with higher organic solvent concentration (Figure 6). In 20% v/v ethanol, the activity was still at 40% of initial activity, while in the presence of isobutanol or n-butanol the activity dropped much quicker with increasing solvent concentrations. Here, less than 6% of the initial enzyme activity was detected at a final concentration of 9% v/v isobutanol or n-butanol after 30 min.

**Figure 7:**
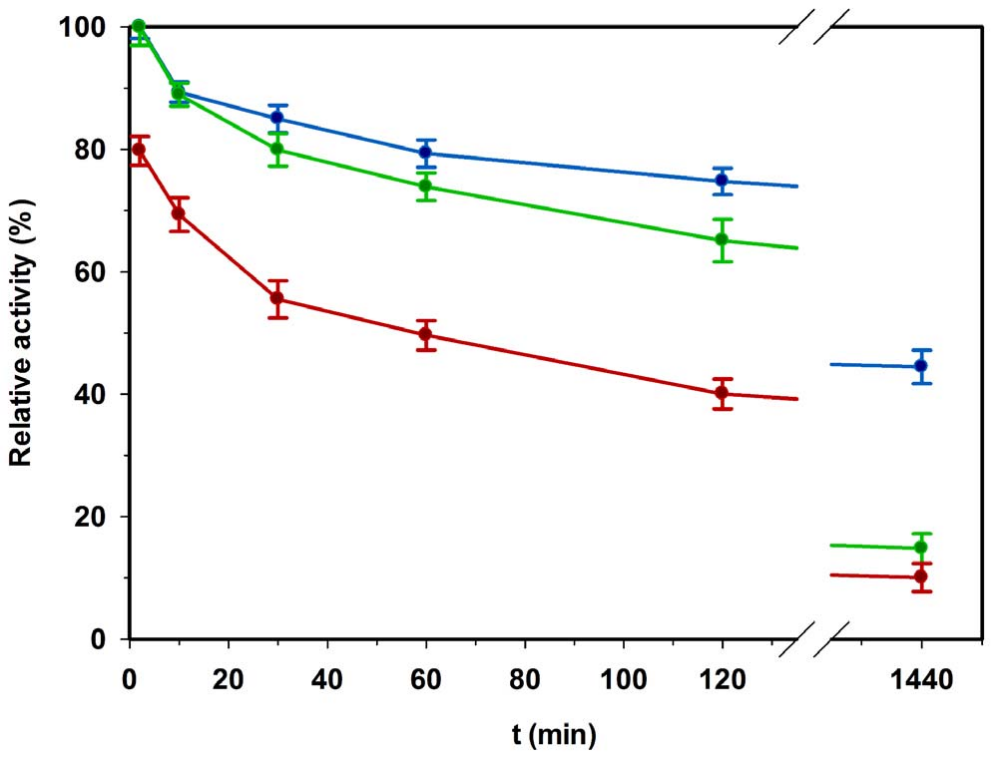
Time course of *Ta*AlDH deactivation by organic solvent and its reactivation. Deactivation of refolded *Ta*AlDH was measured after incubation for indicated time at 50°C in 3% v/v isobutanol (red) or 0.3% v/v isobutanol (blue). Furthermore, samples incubated in 3% v/v isobutanol were also measured after 10-fold dilution to 0.3% v/v isobutanol (green) to test reactivation within 2 min.

To further study the reversibility of inactivation by organic solvents, the time course of activity decrease in the presence of isobutanol was recorded (Figure 7). *Ta*AlDH deactivation by 3% v/v isobutanol at 50°C occurred immediately within 2 min down to 80% of initial activity. After 30 min of *Ta*AlDH deactivation, activity dropped to 55%, but even after 24 h at 3% v/v isobutanol a residual activity of 10% was retained. Slight inactivation was also observed in 0.3% v/v isobutanol leading to loss of ca. 50% activity after 24 h.

Inactivation of enzyme in the absence of isobutanol was similar to that in 0.3% v/v isobutanol (data not shown). Activity could be recovered partially within 2 min when isobutanol was diluted from 3% v/v to 0.3% v/v. After 24 h of inactivation this effect declined substantially indicating an increasing irreversibility of inactivation with enduring incubation. Reactivation times longer than 2 min (10 and 30 min) after dilution from 3% v/v to 0.3% v/v isobutanol did not lead to a further activity increase (data not shown), indicating that reactivation was very fast.

### Characterization of *Ta*AlDH Structural Stability

Ellipticity from soluble and refolded *Ta*AlDH was positive between 260 nm and 280 nm indicating the presence of tertiary structure in the protein (Figure 4B). The signal decreased steeply below 260 nm, which is characteristic for α-helical secondary structures of the folded variants. In contrast, ellipticity from *Ta*AlDH in 6 M GdmCl was close to zero between 260 nm and 280 nm and also below 260 nm indicating complete loss of tertiary and α-helical secondary structures, respectively.

**Figure 8:**
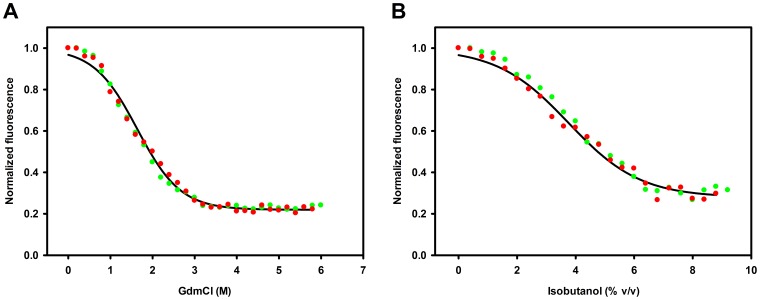
Fluorescence analysis of unfolding and refolding of *Ta*AlDH in the presence of A) GdmCl (25°C) and B) isobutanol (40°C). The fluorescence emissions of *Ta*AlDH at λ = 330 nm were monitored upon excitation at λ_max_ = 280 nm. Data were collected for protein unfolding (red symbols) and refolding (green symbols) at indicated concentrations of GdmCl or isobutanol. The transition curve for protein unfolding is presented as the best fit using nonlinear regression (black curve).

To determine thermodynamic stability, GdmCl and isobutanol induced unfolding of *Ta*AlDH was examined by fluorescent measurements (Figure 8). To test the full reversibility of enzyme unfolding samples of folded as well as fully unfolded *Ta*AlDH were diluted into different concentrations of isobutanol or GdmCl. Identical fluorescence values at respective isobutanol or GdmCl concentrations were obtained indicating complete reversibility under assay conditions (Figure 8). Due to poor solubility of isobutanol in water (formation of streaks and separate organic phase above 9% v/v isobutanol), no well resolved baseline for unfolded *Ta*AlDH in isobutanol could be obtained. Constant fluorescence emissions were assumed for folded *Ta*AlDH in 0.0–0.4% v/v isobutanol and 0.0–0.2 M GdmCl as well as for unfolded *Ta*AlDH in 4.0–5.8 M GdmCl and 8.0–8.8% v/v isobutanol. From the fitted GdmCl plot, the parameter of cooperativeness m_GdmCl_ = −4.8±0.2 kJ/(mol·M) and the free Gibbs energy change ΔG^0^_D_ = 7.9±0.4 kJ/mol was calculated for *Ta*AlDH. Fitting the plot for unfolding in isobutanol, the calculations indicated m_isobutanol_ = −2.1±0.1 kJ/(mol·M) and ΔG^0^_D_ = 8.0±0.5 kJ/mol for *Ta*AlDH. Accordingly, more than 95% *Ta*AlDH was unfolded in concentrations exceeding 3.2 M GdmCl or 7.6% v/v isobutanol.

## Discussion

Synthetic Cascade Biomanufacturing can become a powerful technology, when the right enzymes are available, combining correct specificity, activity and stability. *Ta*AlDH is such an enzyme for a key reaction step in the synthetic 4-enzyme glycolysis for the conversion of glucose to pyruvate. Being reported to be active with NADP^+^, we found the enzyme to also accept NAD^+^ under technically relevant conditions (cofactor >1 mM). Most importantly, *Ta*AlDH has very high substrate specificity. Due to its thermophilic origin *Ta*AlDH has an acceptable thermostability. The optimum temperature was reported to be 50–55°C [36]. Thermostability is often correlated with stability in organic solvents [37], [38], however in this respect, *Ta*AlDH does not show a remarkable tolerance. At 5% ethanol and 2% isobutanol or n-butanol a significant decrease in activity could be observed. An isobutanol induced unfolding transition accordingly revealed a thermodynamic stability (ΔG^0^ of folding) of −8.0±0.5 kJ/mol at 40°C, based on a 2-state model [30] and reversible unfolding, an assumption which was shown to be valid in this case (Figure 8). Stability measurement by GdmCl induced unfolding transition gave a similar value of −7.9±0.4 kJ/mol. Generally, this value is rather low for an enzyme of thermophilic origin [37], [39], showing that there is a large potential for optimization [40], [41]. To stabilize proteins in organic solvents, free energies of intramolecular interactions have to be increased relative to those for protein-solvent interactions. In this regard, Arnold suggested rules for protein stabilization. Exchange of amino acids should satisfy most of protein hydrogen bonds and increase the number of cross-linked salt bridges to stabilize protein substructures and to raise the unfolded state energy, respectively. Furthermore, the increase of free energy of protein unfolding could be accomplished by a more compact packing of hydrophobic side chains, e.g. replacing threonine by smaller hydrophobic amino acids like valine or isoleucine [40], [42]. These considerations could help in stabilizing the enzyme by protein engineering. First experiments are underway here.

Recombinant enzyme production using *E. coli* as host is common standard and was done previously for *Ta*AlDH, however, no data on yields were given [35]. In our hands only very small amounts of enzyme could be obtained in soluble form, the major part of enzyme production was directed into inclusion bodies. This is not uncommon when working with enzymes of archaeal origin [43], [44]. All attempts failed to increase the yield of soluble protein by altering culture conditions. Soluble, active enzyme in large amounts could, however, successfully be obtained by *in vitro* refolding from inclusion bodies. The catalytic properties of soluble and refolded *Ta*AlDH were similar (0.3 U/mg) and spectroscopic analysis showed them to be identical (Figure 4). Protein refolding from inclusion bodies is working for small-scale enzyme production [45] and so *Ta*AlDH was obtained in high yields as a functional enzyme. However, the method is very time consuming in comparison to soluble purification. Furthermore, the use of large amounts of buffers and the decrease in wear and tear of machines by chaotropic agents boost enzyme production costs. Thus, for large-scale industrial biocatalysis, *Ta*AlDH needs to be more efficiently and recombinantly produced in soluble form, another target for enzyme engineering [46].

An objective for further studies on *Ta*AlDH would be the examination of stereo selectivity. Although enantiomerically pure d-glycerate is already applied today for synthesis of non-chiral sugar acid derivatives [47], [48], [49], the enzyme may be useful for future applications in biocatalytic processes for chiral chemicals [50]. For L-glyceraldehyde production by a glycerol dehydrogenase from glycerol, *Ta*AlDH could serve as cofactor recycling enzyme and would simultaneously oxidize d-glyceraldehyde out of a racemic mixture for L-glyceraldehyde enrichment [51].

This study emphasizes *Ta*AlDH as a valuable option when enzyme specialists are needed for new biocatalytic production of chemicals.

## Supporting Information

Figure S1 ***Ta*** **AlDH homology model, based on the crystal structure of betaine aldehyde dehydrogenase (betB) from** ***Staphylococcus aureus*** **(RCSB PDB ID 3FG0).** α-Helices and β-sheets are colored red and yellow, respectively. (Last update 25.04.2013).
(TIF)

## References

[1] Atsumi S, Wu TY, Machado IMP, Huang WC, Chen PY, et al (2010) Evolution, genomic analysis, and reconstruction of isobutanol tolerance in *Escherichia coli* . Molecular Systems Biology 6: 11. 10.1038/msb.2010.98 PMC3018172 21179021

[2] Brynildsen MP, Liao JC (2009) An integrated network approach identifies the isobutanol response network of *Escherichia coli* . Molecular Systems Biology 5: 13. 10.1038/msb.2009.34 PMC2710865 19536200

[3] Guterl J-K, Sieber V (2013) Biosynthesis “debugged”: Novel bioproduction strategies. Engineering in Life Sciences 13: 4–18.

[4] Algar EM, Scopes RK (1985) Studies on cell-free metabolism - Ethanol-production by extracts of *Zymomonas mobilis* . Journal of Biotechnology 2: 275–287.

[5] Zhang YHP, Evans BR, Mielenz JR, Hopkins RC, Adams MWW (2007) High-Yield Hydrogen Production from Starch and Water by a Synthetic Enzymatic Pathway. Plos One 2: 6. 10.1371/journal.pone.0000456 PMC1866174 17520015

[6] Ye XH, Wang YR, Hopkins RC, Adams MWW, Evans BR, et al (2009) Spontaneous High-Yield Production of Hydrogen from Cellulosic Materials and Water Catalyzed by Enzyme Cocktails. ChemSusChem 2: 149–152. 19185036 10.1002/cssc.200900017

[7] Zhang YHP, Myung S, You C, Zhu Z, Rollin JA (2011) Toward low-cost biomanufacturing through *in vitro* synthetic biology: bottom-up design. Journal of Materials Chemistry 21: 18877–18886.

[8] You C, Zhang YHP (2012) Cell-Free Biosystems for Biomanufacturing. Springer Berlin Heidelberg. 1–31.

[9] Guterl J-K, Garbe D, Carsten J, Steffler F, Sommer B, et al (2012) Cell-Free Metabolic Engineering: Production of Chemicals by Minimized Reaction Cascades. ChemSusChem 5: 2165–2172. 23086730 10.1002/cssc.201200365

[10] Atsumi S, Hanai T, Liao JC (2008) Non-fermentative pathways for synthesis of branched-chain higher alcohols as biofuels. Nature 451: 86–89. 18172501 10.1038/nature06450

[11] Pandey A, Nigam P, Soccol CR, Soccol VT, Singh D, et al (2000) Advances in microbial amylases. Biotechnology and Applied Biochemistry 31: 135–152. 10744959 10.1042/ba19990073

[12] Janker-Obermeier I, Sieber V, Faulstich M, Schieder D (2012) Solubilization of hemicellulose and lignin from wheat straw through microwave-assisted alkali treatment. Industrial Crops and Products 39: 198–203.

[13] Kolb M, Sieber V, Amann M, Faulstich M, Schieder D (2012) Removal of monomer delignification products by laccase from *Trametes versicolor* . Bioresource Technology 104: 298–304. 22176974 10.1016/j.biortech.2011.11.080

[14] Rohowsky B, Häßler T, Gladis A, Remmele E, Schieder D, et al. (2012) Feasibility of simultaneous saccharification and juice co-fermentation on hydrothermal pretreated sweet sorghum bagasse for ethanol production. Applied Energy.

[15] Chenault HK, Whitesides GM (1987) Regeneration of Nicotinamide Cofactors for use in Organic Synthesis. Applied Biochemistry and Biotechnology 14: 147–197. 3304160 10.1007/BF02798431

[16] Guagliardi A, Martino M, Iaccarino I, Rosa MD, Rossi M, et al (1996) Purification and Characterization of the Alcohol Dehydrogenase from a Novel Strain of *Bacillus stearothermophilus* Growing at 70°C. The International Journal of Biochemistry & Cell Biology 28: 239–246. 8729010 10.1016/1357-2725(95)00138-7

[17] Jankowski MD, Henry CS, Broadbelt LJ, Hatzimanikatis V (2008) Group Contribution Method for Thermodynamic Analysis of Complex Metabolic Networks. Biophysical Journal 95: 1487–1499. 18645197 10.1529/biophysj.107.124784 PMC2479599

[18] Rodriguez-Zavala JS, Allali-Hassani A, Weiner H (2006) Characterization of *E. coli* tetrameric aldehyde dehydrogenases with atypical properties compared to other aldehyde dehydrogenases. Protein Science 15: 1387–1396. 16731973 10.1110/ps.052039606 PMC2242541

[19] Perozich J, Kuo I, Wang BC, Boesch JS, Lindahl R, et al (2000) Shifting the NAD/NADP preference in class 3 aldehyde dehydrogenase. European Journal of Biochemistry 267: 6197–6203. 11012673 10.1046/j.1432-1327.2000.01697.x

[20] Yin SJ, Liao CS, Wang SL, Chen YJ, Wu CW (1989) Kinetic evidence for human liver and stomach aldehyde dehydrogenase-3 representing an unique class of isozymes. Biochemical Genetics 27: 321–331. 2803227 10.1007/BF00554167

[21] Studier FW (2005) Protein production by auto-induction in high-density shaking cultures. Protein Expression and Purification 41: 207–234. 15915565 10.1016/j.pep.2005.01.016

[22] Neubauer P, Haggstrom L, Enfors SO (1995) Influence of substrate oscillations on acetate formation and growth-yield in *Escherichia coli* glucose-limited fed-batch cultivations. Biotechnology and Bioengineering 47: 139–146. 18623386 10.1002/bit.260470204

[23] Holzinger A, Phillips KS, Weaver TE (1996) Single-step purification solubilization of recombinant proteins: Application to surfactant protein B. Biotechniques 20: 804–806, 808. 10.2144/96205bm16 8723923

[24] Laemmli UK (1970) Cleavage of Structural Proteins during the Assembly of the Head of Bacteriophage T4. Nature 227: 680–685. 5432063 10.1038/227680a0

[25] Bradford MM (1976) Rapid and Sensitive Method for Quantification of Microgram Quantities of Protein Utilizing Principle of Protein-Dye Binding. Analytical Biochemistry 72: 248–254. 942051 10.1016/0003-2697(76)90527-3

[26] Gasteiger E, Hoogland C, Gattiker A, Duvaud Se, Wilkins MR, et al. (2005) Protein identification and analysis tools on the ExPASy server. 571–607. 10.1385/1-59259-584-7:531 10027275

[27] Schmid FX (1989) Spectral methods of characterizing protein conformation and conformational changes. In: Creighton TE, editor. Protein Structure: a Practical Approach. Oxford: IRL Press. 251–285 p.

[28] Kelly SM, Jess TJ, Price NC (2005) How to study proteins by circular dichroism. Biochimica et Biophysica Acta (BBA) - Proteins and Proteomics 1751: 119–139. 16027053 10.1016/j.bbapap.2005.06.005

[29] Whitmore L, Wallace BA (2008) Protein secondary structure analyses from circular dichroism spectroscopy: Methods and reference databases. Biopolymers 89: 392–400. 17896349 10.1002/bip.20853

[30] Santoro MM, Bolen DW (1988) Unfolding free energy changes determined by the linear extrapolation method. 1. Unfolding of phenylmethanesulfonyl α-chymotrypsin using different denaturants. Biochemistry 27: 8063–8068. 3233195 10.1021/bi00421a014

[31] Kelley LA, Sternberg MJE (2009) Protein structure prediction on the Web: a case study using the Phyre server. Nature Protocols 4: 363–371. 19247286 10.1038/nprot.2009.2

[32] Sørensen HP, Sperling-Petersen HU, Mortensen KK (2003) Production of recombinant thermostable proteins expressed in *Escherichia coli*: completion of protein synthesis is the bottleneck. Journal of Chromatography B 786: 207–214. 10.1016/s1570-0232(02)00689-x 12651016

[33] Kamionka M (2011) Engineering of Therapeutic Proteins Production in *Escherichia coli* . Current Pharmaceutical Biotechnology 12: 268–274. 21050165 10.2174/138920111794295693 PMC3179032

[34] Kaur J, Sharma R (2006) Directed evolution: An approach to engineer enzymes. Critical Reviews in Biotechnology 26: 165–199. 16923533 10.1080/07388550600851423

[35] Reher M, Schönheit P (2006) Glyceraldehyde dehydrogenases from the thermoacidophilic euryarchaeota *Picrophilus torridus* and *Thermoplasma acidophilum*, key enzymes of the non-phosphorylative Entner-Doudoroff pathway, constitute a novel enzyme family within the aldehyde dehydrogenase superfamily. Febs Letters 580: 1198–1204. 16458304 10.1016/j.febslet.2006.01.029

[36] Jung JH, Lee SB (2006) Identification and characterization of *Thermoplasma acidophilum* glyceraldehyde dehydrogenase: a new class of NADP^+^-specific aldehyde dehydrogenase. Biochemical Journal 397: 131–138. 16566751 10.1042/BJ20051763 PMC1479753

[37] Vieille C, Zeikus GJ (2001) Hyperthermophilic Enzymes: Sources, Uses, and Molecular Mechanisms for Thermostability. Microbiology and Molecular Biology Reviews 65: 1–43. 11238984 10.1128/MMBR.65.1.1-43.2001 PMC99017

[38] Wu X, Zhang C, Orita I, Imanaka T, Fukui T, et al. (2013) Thermostable alcohol dehydrogenase from *Thermococcus kodakarensis* KOD1 for enantioselective bioconversion of aromatic secondary alcohols. Applied and Environmental Microbiology. 10.1128/AEM.03873-12 PMC3623261 23354700

[39] Consalvi V, Chiaraluce R, Giangiacomo L, Scandurra R, Christova P, et al (2000) Thermal unfolding and conformational stability of the recombinant domain II of glutamate dehydrogenase from the hyperthermophile *Thermotoga maritima* . Protein Engineering 13: 501–507. 10906345 10.1093/protein/13.7.501

[40] Arnold FH (1988) Protein design for non-aqueous solvents. Protein Engineering 2: 21–25. 3253733 10.1093/protein/2.1.21

[41] Sieber V, Pluckthun A, Schmid FX (1998) Selecting proteins with improved stability by a phage-based method. Nature Biotechnology 16: 955–960. 10.1038/nbt1098-955 9788353

[42] Cowan DA (1997) Thermophilic proteins: stability and function in aqueous and organic solvents. Comparative biochemistry and physiology Part A, Physiology 118: 429–438. 10.1016/s0300-9629(97)00004-2 9406427

[43] Kim S, Lee SB (2008) Soluble expression of archaeal proteins in *Escherichia coli* by using fusion-partners. Protein Expression and Purification 62: 116–119. 18657619 10.1016/j.pep.2008.06.015

[44] Kube J, Brokamp C, Machielsen R, Oost J, Maerkl H (2006) Influence of temperature on the production of an archaeal thermoactive alcohol dehydrogenase from *Pyrococcus furiosus* with recombinant *Escherichia coli* . Extremophiles 10: 221–227. 16463078 10.1007/s00792-005-0490-z

[45] Dashivets T, Wood N, Hergersberg C, Buchner J, Haslbeck M (2009) Rapid Matrix-Assisted Refolding of Histidine-Tagged Proteins. ChemBioChem 10: 869–876. 19235820 10.1002/cbic.200800697

[46] Romero PA, Arnold FH (2009) Exploring protein fitness landscapes by directed evolution. Nature Reviews Molecular Cell Biology 10: 866–876. 19935669 10.1038/nrm2805 PMC2997618

[47] Liu Y, Koh CMJ, Sun L, Ji L (2011) Tartronate Semialdehyde Reductase Defines a Novel Rate-Limiting Step in Assimilation and Bioconversion of Glycerol in *Ustilago maydis* . Plos One 6: e16438. 21305026 10.1371/journal.pone.0016438 PMC3031564

[48] Hedrick JL, Sallach HJ (1964) The nonoxidative decarboxylation of hydroxypyruvate in mammalian systems. Archives of Biochemistry and Biophysics 105: 261–269. 14186730 10.1016/0003-9861(64)90007-4

[49] Ruszczycky MW, Anderson VE (2004) Tartrate dehydrogenase reductive decarboxylation: stereochemical generation of diastereotopically deuterated hydroxymethylenes. Bioorganic Chemistry 32: 51–61. 14700562 10.1016/j.bioorg.2003.09.006

[50] Ogawa J, Shimizu S (2002) Industrial microbial enzymes: their discovery by screening and use in large-scale production of useful chemicals in Japan. Current Opinion in Biotechnology 13: 367–375. 12323360 10.1016/s0958-1669(02)00331-2

[51] Richter N, Neumann M, Liese A, Wohlgemuth R, Eggert T, et al (2009) Characterisation of a Recombinant NADP-Dependent Glycerol Dehydrogenase from *Gluconobacter oxydans* and its Application in the Production of L-Glyceraldehyde. ChemBioChem 10: 1888–1896. 19579248 10.1002/cbic.200900193

[52] Yamanaka Y, Kazuoka T, Yoshida M, Yamanaka K, Oikawa T, et al (2002) Thermostable aldehyde dehydrogenase from psychrophile, *Cytophaga* sp. KUC-1: enzymological characteristics and functional properties. Biochemical and Biophysical Research Communications 298: 632–637. 12419301 10.1016/s0006-291x(02)02523-8

[53] Kato T, Miyanaga A, Kanaya S, Morikawa M (2010) Gene cloning and characterization of an aldehyde dehydrogenase from long-chain alkane-degrading *Geobacillus thermoleovorans* B23. Extremophiles 14: 33–39. 19787414 10.1007/s00792-009-0285-8

[54] Imanaka T, Ohta T, Sakoda H, Widhyastuti N, Matsuoka M (1993) Cloning, nucleotide sequence, and efficient expression of the gene coding for thermostable aldehyde dehydrogenase from *Bacillus stearothermophilus*, and characterization of the enzyme. Journal of Fermentation and Bioengineering 76: 161–167.

