# Supplementary figures and images for: Refolding of a Thermostable Glyceraldehyde Dehydrogenase for Application in Synthetic Cascade Biomanufacturing
Source: PLoS One. 2013 Jul 24;8(7):e70592. doi: 10.1371/journal.pone.0070592 (PMC3722153; doi:10.1371/journal.pone.0070592)

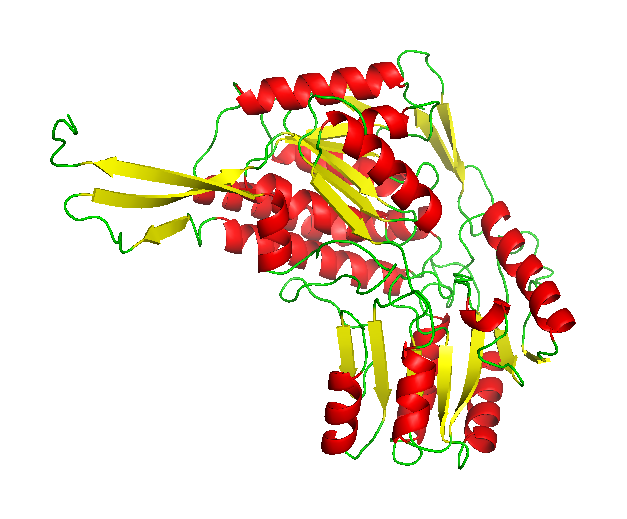

Supplement: Figure S1 — Ta AlDH homology model, based on the crystal structure of betaine aldehyde dehydrogenase (betB) from Staphylococcus aureus (RCSB PDB ID 3FG0). α-Helices and β-sheets are colored red and yellow, respectively. (Last update 25.04.2013). (TIF) [file pone.0070592.s001.tif]
